# Encephalopathy Associated with Severe Cytomegalovirus Infection in an Immunocompetent Young Woman

**DOI:** 10.1155/2021/5589739

**Published:** 2021-06-04

**Authors:** Syuichi Tetsuka, Tomohiro Suzuki, Tomoko Ogawa, Ritsuo Hashimoto, Hiroyuki Kato

**Affiliations:** Department of Neurology, International University of Health and Welfare Hospital, 537-3 Iguchi, Nasushiobara, Tochigi 329-2763, Japan

## Abstract

Primary cytomegalovirus (CMV) infection in healthy young adults is usually an asymptomatic or mononucleosis-like syndrome, whereas in immunocompromised patients, CMV can cause significant disease. In this study, we report an unusual case of primary CMV infection wherein the patient, an immunocompetent 21-year-old woman, presented severe encephalopathy, acute hepatitis, retinitis, and reactivation of latent Epstein–Barr virus. She developed confusion, high fever, headache, and tonic-clonic seizures. Brain magnetic resonance imaging showed high-intensity lesions in the medial temporal lobe and basal ganglia. Liver dysfunction was observed, and abdominal computed tomography revealed splenohepatomegaly. After fundus findings, the patient was diagnosed with CMV retinitis. Upon admission, she was treated with intravenous acyclovir and steroid pulse therapy. Considering both her serious clinical condition and elevated serum levels of interleukin-6, we speculated that her condition was similar to cytokine-storm-induced encephalopathy. On day 2 after admission, she showed prompt recovery from these clinical manifestations. Since blood CMV pp65 antigenemia was found to be positive, we administered ganciclovir for 2 weeks. On the basis of her clinical manifestations and the presence of blood CMV DNA and CMV pp65 antigenemia along with IgM kinetics, we finally diagnosed this patient with severe primary CMV infection. She left our hospital without sequelae 20 days after admission. The incidence of severe CMV disease in immunocompetent young adults might be higher than previously recognized. Noninvasive testing for CMV (such as CMV pp65 antigenemia and CMV DNAemia) is widely available and can help early diagnosis. Short-term glucocorticoid therapy might be beneficial in the treatment of encephalopathy in the early stages of primary CMV infection. Considering such a background, clinicians should keep severe primary CMV infection in mind as a differential diagnosis in the clinical setting.

## 1. Introduction

Cytomegalovirus (CMV), which establishes a lifelong latency period after primary infection, is a herpes virus that is present everywhere with a worldwide seroprevalence ranging from 45% to 100% [1]. In the past, most people were first infected in the early childhood, but the age of first infection has been rising as the sanitary environment improves in Japan. CMV causes serious diseases in immunocompromised patients and results in significant morbidity and mortality through reactivation of the latent virus or primary infection [2]. Advances in diagnostic technology have revealed that CMV is a common opportunistic infection in fetuses, allograft recipients, bone marrow transplant patients, solid organ transplants, hematopoietic stem cell transplants, and acquired immunodeficiency syndrome (AIDS) patients [3]. Primary CMV infection in healthy young adults is usually asymptomatic in 80% of immunocompetent individuals, and only approximately 20% of patients have mild influenza- or mononucleosis-like symptoms, which include fever, rhinitis and pharyngitis, headache, arthralgia and myalgia, and physical exhaustion [4]. Therefore, the onset of CMV encephalopathy or encephalitis in immunocompetent patients is thought to be extremely rare. However, recently, it seems that the incidence of severe manifestations of CMV infection in immunocompromised individuals has been recognized to be much more common than before. Thus, severe CMV infections, such as encephalopathy, hepatitis, retinitis, gastrointestinal disorders, cardiovascular diseases, pneumonia, and hematological abnormalities in immunocompetent adults, might become a condition that should be considered as one of the differential diagnoses in many future clinical settings [5]. We present a case of an immunocompetent young adult who recovered promptly from severe encephalopathy, acute hepatitis, and retinitis caused by primary CMV infection.

## 2. Case Presentation

**Figure 1:**
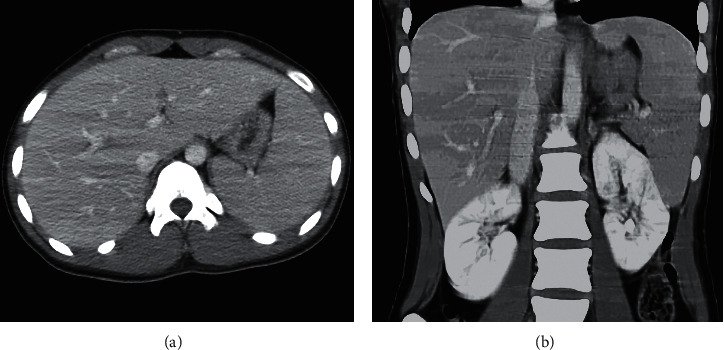
Abdominal contrast-enhanced CT upon admission. (a) Axial view; (b) Coronal view. There is splenohepatomegaly.

**Figure 2:**
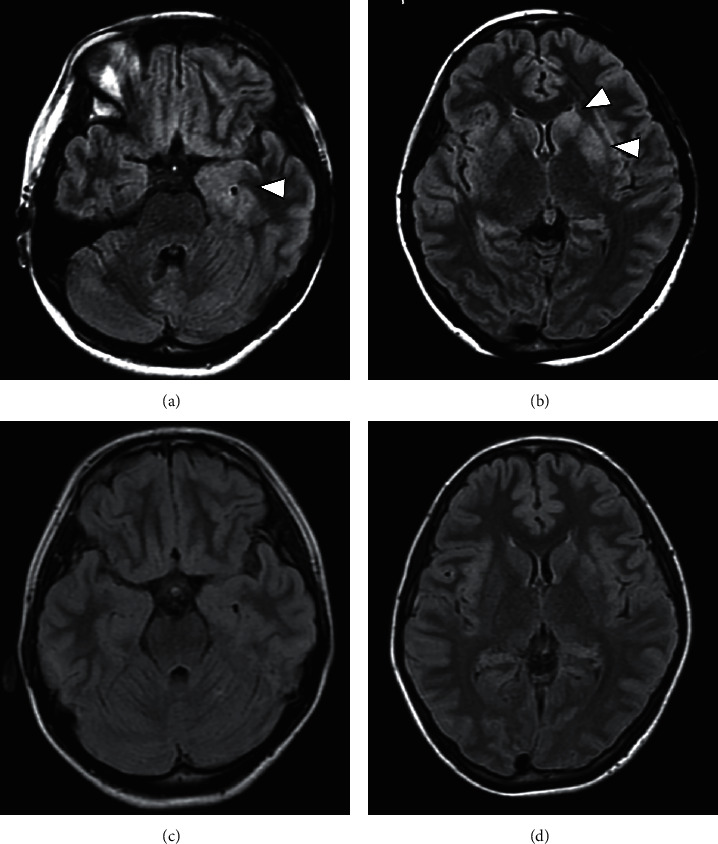
Brain MRI studies at the onset. Axial view of fluid-attenuated inversion recovery (FLAIR) images showing high-intensity lesions in the left medial temporal lobe (a), bilateral caudate nucleus, and left lentiform nucleus (b). (c), (d) MRI studies on the second day of follow-up. FLAIR images show a normal signal in the medial temporal lobe and basal ganglia and no abnormal lesion. Arrowheads indicate high-intensity lesions.

A 21-year-old woman presented to the emergency room at our hospital with a history of confusion, high fever, and headache. Six days before emergency consultation, she had fever in the 38°C range and had a loss of appetite. She had a history of mumps at the age of seven. Other than that, there was nothing remarkable about her medical or medication history. After admission on the same day, her level of consciousness worsened, and she could barely open her eyes when called, but she did not speak, did not get the instructions, and did not move her limbs (Glasgow Coma Scale; E3V1M1). In addition, tonic-clonic seizures that lasted for a few minutes began to occur. Her initial vital signs were as follows: heart rate, 128 beats/min; respiratory rate, 24 breaths/min; blood pressure, 130/81 mmHg; body temperature, 39.9°C; and oxygen saturation, 94% with room air. Neurological examination revealed no neck stiffness and no other gross focal neurological deficits. Results of laboratory investigations are shown in Table 1. Her white blood cell count and C-reactive protein concentration were 11530/*µ*l (neutrophils, 47.0%; lymphocytes, 27.0%; monocytes, 3.0%; and atypical lymphocytes, 23.0%) and 3.34 mg/L, respectively. Also, many atypical lymphocytes were observed in the peripheral blood smear. Ferritin levels were mildly elevated in the serum (68 ng/ml). Liver dysfunction was observed with an elevated aspartate aminotransferase (AST) level (116 IU/L) and an elevated alanine aminotransferase (ALT) level (183 IU/L). Serum creatinine and electrolyte concentrations were normal. Serum levels of interleukin-6 (IL-6) were elevated to 10.3 pg/ml (normal range, <4.0 pg/ml). Both nasopharyngeal swab tests for SARS-CoV-2 RNA and influenza antigen (A and B) were negative results. Based on the presence of liver dysfunction by laboratory investigations at admission, abdominal computed tomography (CT) was performed. An abdominal contrast-enhanced CT revealed a splenohepatomegaly but no sign of neoplasia (Figures 1(a) and 1(b)). Cerebrospinal fluid (CSF) analysis showed lymphocyte-dominant pleocytosis with 186 nuclear cells/mm^3^ (180 mononuclear cells/mm^3^ and 6 polymorphonuclear cells/mm^3^) and protein concentration (41 mg/dL) and glucose concentration (96 mg/dL, with a serum glucose concentration of 140 mg/dL). Fluid-attenuated inversion recovery (FLAIR) images of brain magnetic resonance imaging (MRI) showed high-intensity lesions at the left medial temporal lobe, bilateral caudate nucleus, and left lentiform nucleus (Figures 2(a) and 2(b)). Based on the patient's clinical findings and the results of various laboratory and imaging tests, we diagnosed her with possible meningoencephalitis, including herpes simplex virus encephalitis. On the day of admission, the patient was started on meningoencephalitis treatment with acyclovir (10 mg/kg/IV q8h), ceftriaxone sodium (2 g IV q12h), and fosphenytoin sodium (IV q24h) as antiepileptic drugs and steroid pulse therapy with high doses of intravenous methylprednisolone (1,000 mg) for 3 days.

**Figure 3:**
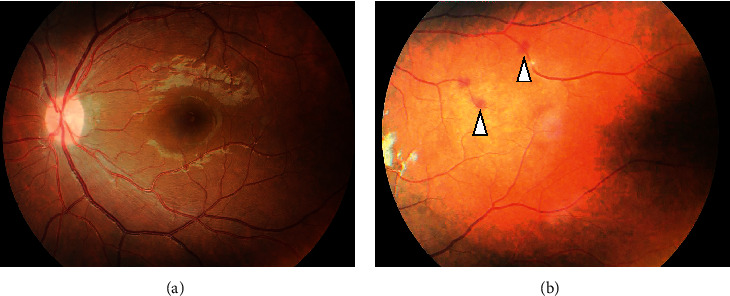
Fundus appearance on admission. There is white punctate exudate around the macula (a) and petechiae were observed (b) in the retina of the left eye. Arrowheads indicate petechiae.

On the second day of admission, she became afebrile, was able to speak, and was shown to improve her disturbance of consciousness. Serial FLAIR images of brain MRI showed no abnormality and the improvement of previous MRI findings (Figures 2(c) and 2(d)). In the results of laboratory investigations on admission, blood CMV pp65 antigenemia assay was found to be positive (17 pp65-positive cells per 5 × 10^5^ cells) and herpes simplex virus (HSV) DNA was negative in the CSF. Thus, we suspected that encephalopathy was caused by CMV and started to administer ganciclovir (5 mg/kg/IV q12h) instead of acyclovir. As the possibility of CMV infection increased, she consulted an ophthalmologist. As fundus findings, white punctate exudate and petechiae were observed in the retina of the left eye, and a diagnosis of CMV retinitis and uveitis was made (Figures 3(a) and 3(b)). However, symptoms such as decreased visual acuity and visual field impairment had not yet been developed. After that, her general condition recovered rapidly, and after two weeks, she was able to walk and talk normally. In other results of laboratory investigations on admission, blood CMV DNA was detected by polymerase chain reaction (PCR) (1.6 × 10^3^ copies/ml), but CSF CMV DNA was negative (Table 1). The test for Epstein–Barr virus (EBV) DNA in blood was also positive. Anti-CMV IgM antibody levels were elevated (8.54 titers), as were anti-CMV IgG antibodies (15.5 titers) (enzyme-linked immunosorbent assay; positive titer >2.0), in the serum. The human immunodeficiency virus (HIV) antibody testing using rapid tests was negative, and HIV RNA was undetectable in the serum. The number of CD4+ cells was normal. Antinuclear antibody, anti-double-stranded DNA antibody, and hepatitis screening results were negative, and serum immunoglobulin levels (IgG, IgM, IgA, and IgE) were within normal limits. No other potential immunosuppressant factors were recognized in the patient in terms of clinical manifestation or laboratory findings. In addition, the IgM antibody levels of various viruses, including EBV, varicella-zoster virus, and mumps virus, were also elevated in serum. After two weeks of administration of ganciclovir, blood CMV pp65 antigenemia assay became negative and the administration of this drug was terminated. In addition, blood CMV DNA was also negative (Table 1). Two weeks after admission, a retest of CMV antibodies showed that IgM was still positive (7.49 titers), but IgG antibody titer was significantly higher (71.0 titers) than before (15.5 titers) (Table 1). Lower serum levels of IL-6 were observed (2.4 pg/ml). Considering the presence of CMV DNA and pp65 antigenemia along with IgM kinetics, CMV infection in the patient was suggestive of a primary infection. During the subsequent course, her liver function improved, and retinitis did not worsen. She recovered 20 days after admission and was able to leave our hospital without sequelae. After 4 months, follow-up laboratory investigations were performed, and blood CMV pp65 antigenemia was confirmed to be negative. The IgM antibody levels of all viruses, including CMV, EBV, varicella-zoster virus, and mumps virus, changed to negative or showed a significant decrease (Table 1). She is on outpatient follow-up and is doing well.

Regarding the relative changes in the patient's IgM and IgG levels over time, CMV IgM four months after admission was observed to decrease to 2.99 titers. Conversely, CMV IgG levels four months after admission were 42.5 titers, which were not significantly different from the values (71.0 titers) two weeks after admission.

## 3. Discussion and Conclusions

We finally diagnosed this patient with severe primary CMV infection, including encephalopathy, acute hepatitis, and retinitis. With regard to encephalopathy, although this patient exhibited central nervous system involvement, CSF CMV DNA was not detected. Therefore, it is unknown whether the CMV invaded the central nervous system directly. It has been reported that the sensitivity and specificity of CSF CMV DNA by the PCR method in the identification of active CMV infection of the central nervous system were 93.3% and 93.7%, respectively, and the sensitivity and specificity of CSF CMV pp65 antigenemia assay were 84.6% and 100%, respectively [6]. In the case of latent CMV infection, CMV DNA is not found in the CSF; thus, the detection of CMV DNA in the CSF is a marker of active infections such as CMV encephalitis. Considering such data with laboratory findings, MRI findings, and clinical presentation, we diagnosed encephalopathy, which indirectly causes central nervous system symptoms, instead of encephalitis. On the other hand, blood CMV DNA and CMV pp65 antigenemia were positive. The liver and retina of this patient were considered to be directly invaded by CMV itself, and she developed acute hepatitis and retinopathy. In CMV infections, if healthy adults in whom serum CMV IgG antibody remains negative are first infected, the patients often present with infectious mononucleosis-like symptoms. Fever, liver dysfunction, cervical lymphadenopathy, hepatosplenomegaly, etc. are the main symptoms, and it is difficult to distinguish them from those of the initial infection with the EBV. Moreover, since blood EBV DNA and serum EBV IgM were positive in this case, it is not possible to determine whether CMV or EBV contributed more to acute encephalopathy and acute hepatitis. But, her retinitis was considered to be most likely due to CMV, which is an involvement specific to CMV infection typically. In any case, she had a very rare condition as an immunocompetent young woman.

Several mechanisms have been proposed as the cause of simultaneous positive IgM for EBV and CMV. Given that blood CMV DNA and EBV DNA were detected in this case they include, it is considered that there is the possibility of the two mechanisms: (1) EBV and CMV superinfection and (2) reactivation of latent EBV caused by transient suppression of cell-mediated immunity or settings of acute systemic stress such as cytokine storm caused by CMV infection [7–9]. Furthermore, anti-Epstein–Barr virus nuclear antigen (EBNA) IgG was persistently positive from the beginning; thus, we could rule out the possibility of primary infection of EBV (Table 1). Serum anti-EBNA IgG is detected months after primary infection, which is the recovery period and is an indicator of the history of the infection. The detections of other viral IgM, including varicella-zoster virus and the mumps virus, are insufficient to diagnose viral reactivation; however, such elevated IgM antibodies are frequently observed as nonspecific heterologous IgM during an acute infection. Thus, we diagnosed the patient with only latent EBV reactivation because the other viruses were only indirectly detected. On the other hand, regarding CMV infection, fluctuation in the CMV IgG titers was observed with CMV IgG titers rising from 15.5 titers on admission to 71.0 titers two weeks after the admission. Furthermore, because the presence of CMV DNA and pp65 antigenemia along with IgM kinetics, which were also observed in this case (Table 1), are indicators of a primary infection, we finally diagnosed this patient with severe primary CMV infection, taking into account her clinical findings. If viral replication is out of control by those immune systems, it can lead to severe organ disease and, in some cases, death, particularly in conditions such as AIDS and organ transplantation. In this case, we could make an early diagnosis by using a CMV pp65 antigenemia test and start treating her with ganciclovir on day 2 after admission, which would have led to a good prognosis for her. Diagnosing CMV diseases, just like a lot of other diseases, is difficult in immunocompetent individuals. Although we initially have suspected her suffering from acute HIV infection, considering her manifestations and results of laboratory tests, the differential diagnosis was ruled out based on the negative blood HIV RNA (Table 1). Severe primary CMV infection has a significant diagnostic challenge, especially in immunocompetent young adults, which might lead to delayed diagnosis and adverse health outcomes.

With regard to her experiencing a rapid improvement in clinical symptoms in a short period after the start of treatment, it is supposed that steroid pulse therapy with high doses of glucocorticoid was remarkably effective and she may have had a condition like the so-called cytokine storm. However, steroid administration also has a reverse impact on CMV infection, taking into account her clinical findings. Steroids aggravate the disease, increase the CMV infection rate, and increase the duration of antiviral therapy in patients at high risk of CMV infection [10]. For example, in transplant recipients, steroid bolus for rejection often leads to CMV reactivation [11]. Therefore, as in this case, we consider that high-dose steroid administration should be maintained for a short period in the early stages of the disease and not for a long period, while ruling out potential immunosuppressive conditions [12, 13]. Acute encephalopathy due to cytokine storm is characterized by high levels of serum IL-6, an inflammatory cytokine that plays an important role in cytokine release syndrome [14]. CMV infection induces IL-6, which might contribute to the pathology of the infection [15]. Also, in this case, high levels of IL-6 were observed before steroid pulse therapy, but after treatment, IL-6 also normalized with improvement in clinical symptoms (Table 1). Following the EBV, CMV is the most frequently reported as an infectious trigger for cytokine storm. CMV-related cytokine storm has a poor prognosis as with most other cytokine storm, and there are no randomized controlled trials for the treatment of virus-induced cytokine storm. The treatment is not limited to antiviral drugs, and the combined use of immunomodulatory therapy as in this case is extremely important for improving the prognosis of such patients.

In conclusion, this case report shows a rapid recovery process and good prognosis from severe CMV encephalopathy due to cytokine storm, acute hepatitis, retinitis, and latent EBV reactivation in an immunocompetent young woman. The incidence of severe CMV disease in immunocompetent young adults may be more than previously recognized. The presence of CMV DNA and pp65 antigenemia along with IgM kinetics can be indicators of a primary infection. The experience suggests that early short-term glucocorticoid therapy might be beneficial in severe cases of primary CMV infection with encephalopathy. Also, noninvasive testing for CMV such as the CMV DNAemia and pp65 antigenemia test is widely available and comes with the possibility of promptly obtaining results; thus, it can help early diagnosis if used appropriately. Considering such a background, clinicians should keep severe primary CMV infection in mind as the differential diagnosis in clinical settings.

## Figures and Tables

**Table 1:** Laboratory investigations

Investigations	Result on admission	Results two weeks after the admission	Results four months after the admission
WBC (/*µ*l), (3500–9700)	11530	7600	6800
Differential count (%)	N 47.0, L 27.0, M 3.0, A 23.0	N 68.0, L 25.4, M 6.6, A 0	N 60.5, L 34.9, M 4.6, A 0
Hemoglobin (g/dl), (11.2–15.2)	12.4	12.2	12.5
Platelet count (×10^9^/L), (14.9–37.9)	22.2	30.3	23.1
CRP (mg/dl), (<0.3)	3.34	0.04	0.04
HBV, HCV serology	Negative		
Serum HIV antibody	Negative		Negative
Blood HIV RNA	Negative		
AST (U/l), (8–38)	116	20	21
ALT (U/l), (4–44)	183	22	16
Glucose (mg/dl), (70–109)	140	158	93
CSF counts (cells/mm^3^)	WBC 186 (N 6, L 180)	WBC 21 (N 0, L 21)	
CSF glucose (mg/dl), (50–75)	96	58	
CSF protein (mg/dl), (15–45)	41	37	
CSF gram stain and culture	Negative		
CSF TB-PCR	Negative		
CSF fungal culture	Negative		
Nasopharynx influenza antigen (A and B)	Negative		
Nasopharynx COVID-19 RNA	Negative		
Blood CMV pp65 antigenemia (0 pp65-positive cells per 5 × 10^5^ cells)	Positive (17)	Negative (0)	Negative (0)
Blood CMV DNA	Positive (1.6 × 10^3^ copies/ml)	Negative	
CSF CMV DNA	Negative		
Serum anti-CMV IgM (<0.8 titers)	Positive (8.54)	Positive (7.49)	Positive (2.99)
Serum anti-CMV IgG (<2.0 titers)	Positive (15.5)	Positive (71.0)	Positive (42.5)
CSF HSV DNA	Negative		
Serum anti-HSV IgM (<0.8 titers)	Negative (0.28)		
Serum anti-HSV IgG (<2.0 titers)	Negative (<2.0)		
Blood EBV DNA	Positive (2.93 log IU/ml)	Negative	
CSF EBV DNA	Negative		
Serum anti-EBNA IgG (<0.5 titers)	Positive (4.0)	Positive (3.1)	Positive (4.3)
Serum anti-VCA-IgM (<0.5 titers)	Positive (12.7)	Positive (8.9)	Positive (1.4)
Serum anti-VCA-IgG (<0.5 titers)	Positive (8.2)	Positive (8.3)	Positive (8.4)
Serum anti-VZV IgM (<0.8 titers)	Positive (1.32)	Positive (1.48)	Negative (0.62)
Serum anti-VZV IgG (<2.0 titers)	Positive (89.0)	Positive (74.0)	Positive (55.6)
Serum antimumps IgM (<0.8 titers)	Positive (2.9)	Positive (2.58)	Borderline (0.82)
Serum antimumps IgG (<2.0 titers)	Positive (47.0)	Positive (39.0)	Positive (23.5)
Serum interleukin-6 (pg/ml), (<4)	10.3	2.4	

Normal ranges are given in parentheses. WBC, white blood cell; N, neutrophils; L, lymphocytes; M, monocytes; A, atypical lymphocytes; CRP, C-reactive protein; AST, aspartate aminotransferase; ALT, alanine aminotransferase; HBV, hepatitis B virus; HCV, hepatitis C virus; HIV, human immunodeficiency virus; CSF, cerebrospinal fluid; TB, tuberculosis; PCR, polymerase chain reaction; CMV, cytomegalovirus; HSV, herpes simplex virus; EBV, Epstein–Barr virus; EBNA, Epstein–Barr virus nuclear antigen; VCA, virus capsid antigen; VZV, varicella-zoster virus; IgM, immunoglobulin M; IgG, immunoglobulin G.

## Data Availability

The data used to support the findings of this study are available from the corresponding author upon request.
